# An Exploratory Spatial Analysis of ALS Incidence in Ireland over 17.5 Years (1995 – July 2013)

**DOI:** 10.1371/journal.pone.0096556

**Published:** 2014-05-27

**Authors:** James Rooney, Mark Heverin, Alice Vajda, Arlene Crampsie, Katy Tobin, Susan Byrne, Anthony Staines, Orla Hardiman

**Affiliations:** 1 Academic Unit of Neurology, Trinity Biomedical Sciences Institute, Trinity College Dublin, Dublin, Ireland; 2 School of Geography, Planning & Environmental Policy, University College Dublin Belfield, Dublin, Ireland; 3 School of Nursing and Human Sciences, Dublin City University, Dublin, Ireland; 4 Department of Neurology, Beaumont Hospital, Beaumont, Ireland; Institute of Health Science, China

## Abstract

**Introduction:**

There has been much interest in spatial analysis of ALS to identify potential environmental or genetically caused clusters of disease. Results to date have been inconclusive. The Irish ALS register has been recently geocoded, presenting opportunity to perform a spatial analysis on national prospectively gathered data of incident cases over an 18-year period.

**Methods:**

1,645 cases of ALS in Ireland from January 1995 to July 2013 were identified from the Irish ALS register. 1,638 cases were successfully geocoded. Census data from four censuses: 1996, 2002, 2006 & 2011 were used to calculate an average population for the period and standardized incidence rates (SIRs) were calculated for 3,355 areas (Electoral Divisions). Bayesian conditional auto-regression was applied to produce smoothed relative risks (RR). These were then mapped for all cases, males & females separately, and those under 55 vs over 55 at diagnosis. Bayesian and linear regression were used to examine the relationship between population density and RR.

**Results:**

Smoothed maps revealed no overall geographical pattern to ALS incidence in Ireland, although several areas of localized increased risk were identified. Stratified maps also suggested localized areas of increased RR, while dual analysis of the relationship between population density and RR of ALS yielded conflicting results, linear regression revealed a weak relationship.

**Discussion:**

In contrast to some previous studies our analysis did not reveal any large-scale geographic patterns of incidence, yet localized areas of moderately high risk were found in both urban and rural areas. Stratified maps by age revealed a larger number of cases in younger people in the area of County Cork - possibly of genetic cause. Bayesian auto-regression of population density failed to find a significant association with risk, however weighted linear regression of post Bayesian smoothed Risk revealed an association between population density and increased ALS risk.

## Introduction

Amyotrophic Lateral Sclerosis (ALS) is a terminal neurodegenerative condition of complex genetic origin, with an annual incidence in Ireland of approximately 2.6 per 100,000 and a lifetime risk of 1∶300[1]. At least 18 Mendelian inherited genes are known to be important in ALS pathogenesis, the most prevalent of which is a hexanucleotide repeat expansion in C9orf72 which accounts for up to 11% of all ALS in Ireland[2]. A larger number of possible susceptibility genes have also been suggested, but many of the reported genes have yet to be confirmed[3]. As ALS is a disease that manifests later in life, genetic factors present at birth are likely to interact with later-life environmental factors. Early observations from Guam[4] and the Kii peninsula of Japan[5], [6] of localised higher incidence rates of ALS generated interest in possible environmental causes, leading to interest in the spatial analysis of ALS incidence[7]–[10]. Since then, the methods of spatial analysis have evolved along with advances in technology, the development of new statistical approaches and with the emergence of geospatial information systems (GIS)[11].

A number of different approaches have been taken in spatial analysis of ALS, however these can be crudely divided into two groups (although some studies have characteristics of both groups). The first group includes studies that have analyzed area based incidence rates – largely as an exploratory hypothesis-generating tool. The second group of studies include those which have implemented formal cluster analysis – either as an exploratory tool, or alternatively to investigate purported clusters. Findings have been conflicting, partly because many of the reported studies have been based on mortality data[9], [12]–[15], which may be subject to incomplete case ascertainment thereby introducing a spatial bias. These data are therefore considered to be less reliable than those derived from prospective data collection, for example via population based register. Only two studies, one from Italy, one from the South-East of England are truly population based and have used a prospective design and case ascertainment, although numbers from the latter study are relatively small. [8], [10].

Whilst most cluster analysis studies have used one or several spatial scan statistics, few studies have employed spatial smoothing. Just two of the studies used Bayesian spatial smoothing[8], [12]. This computationally intensive method, which has become practical with advances in computer hardware and software, has been used extensively to map cancer incidence in Ireland[16]. This method has the advantage of being able to allow for global and local random effects within a conditional auto-regression model[17].

Earlier analysis (1999) of the epidemiology of ALS in Ireland yielded a suggestion of an area of high incidence in the North-West of the country – encompassing the counties of Donegal, Sligo and Leitrim[18]. This finding was significant using crude incidence rates (P = 0.017), however the observation did not meet criteria for statistical significance after standardization (P = 0.07).

This study aims to employ modern spatial analysis methods to produce a high quality map and perform a formal spatial analysis of ALS incidence in Ireland. We have taken a step-by-step approach to employ modern GIS techniques to map ALS in Ireland, focusing in this paper on an exploratory areal visualization of Bayesian smoothed SIR's using a population based cohort from the Irish ALS register.

## Methods

### Ethics Statement

The Irish ALS Register complies with Irish Data protection legislation (1988 and 2003), and has been approved by Beaumont Hospital Ethics Committee (02/28 and 05/49). Approval for the study is from Beaumont hospital ethics committee (05/49). Verbal consent is obtained from all participants for inclusion on the Irish ALS Register. All cases have written documentation of verbal approval. The Irish Data Protection Commissioner has provided written confirmation of compliance with Irish data protection legislation. This approval is on file with the local IRB.

### Data Sources

**Figure 1:**
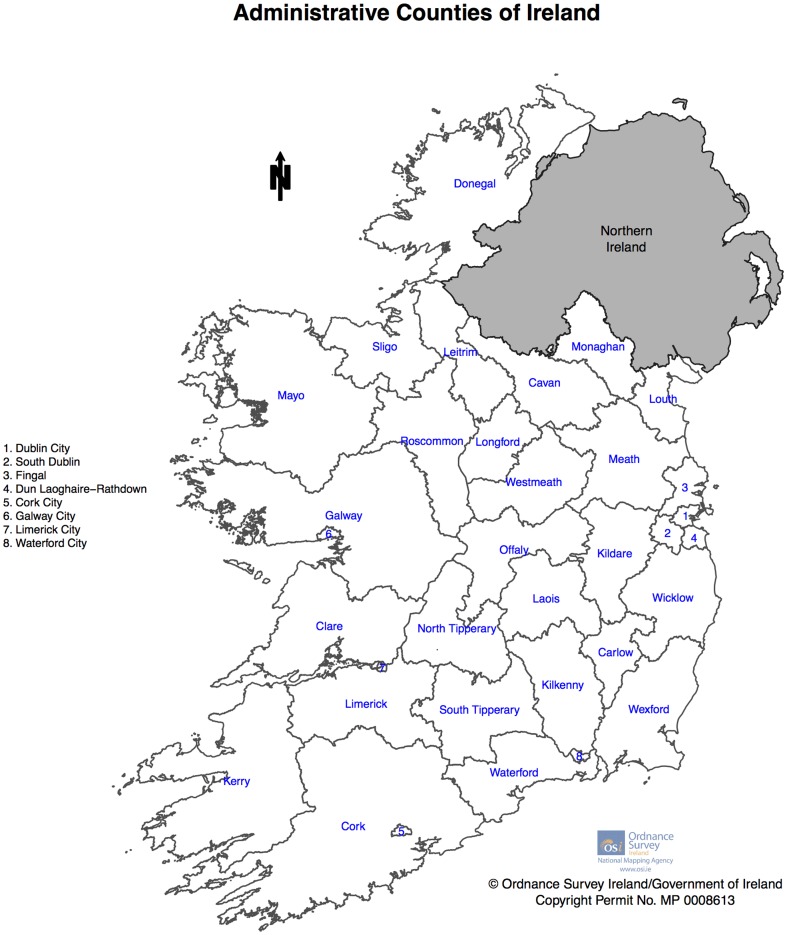
Administrative Counties of Ireland.

Ireland is divided into 34 administrative areas – depicted in Figure 1. These larger administrative areas, are further subdivided into 3,409 Electoral Divisions (for the 2011 Census). Boundary files for the Electoral Divisions were obtained from the Ordnance Survey of Ireland[19]. Data on the Irish population was obtained from the Irish Central Statistics Office (CSO). Census data by Electoral Division were available online for the years 2002, 2006 and 2011 [20]–[23]. For the census year 1996, 5-year age group data was only available for larger areas, however total male and female data was available by electoral division[24] and these were used to apportion the larger area 5-year age group data to electoral divisions on a proportional basis. Average population per Electoral Division by gender per 5 year age group across the four censuses was then calculated.

Patient data was obtained from the Irish ALS register which has been extensively described [1], [18]. All eligible cases (diagnosed between 1^st^ January 1995 and 31^st^ July 2013) were geocoded using their address at time of diagnosis, (X-Y co-ordinates of latitude and longitude, World Geodetic System 84). As Ireland is one of the few developed countries in the world that does not use postcodes, geocodes were produced manually using the Ordnance Survey Ireland website[25]. Some addresses were more suited to precise geocoding than others - when the address contains a house-name or street number a relatively precise geocode could be generated compared to an address that referred only to a townland. In these cases the central point of the townland was taken as the address. However, even rural addresses can be geocoded to an accuracy of within a few hundred metres of the house, depending on the size of the townland. Each search result was inspected visually to check that the parameters of the address (townland, town, county) were consistent with the search result.

### Calculation of Standardized Incidence Rates

Indirect standardization by 5 year age group and sex was employed. Nationwide incidence rates per 5 year age group aged 20 and over, and by gender were calculated. These nationwide incidence rates were then applied to the average population data per Electoral Division to calculate the expected number of cases over the period of the study. For any cases missing age at diagnosis a correction factor was used to distribute each case across all age groups according to the overall age distribution. This ensured that observed and expected case totals were equal. This was also preferable to excluding those cases as that would have led to large localized errors when calculating ED SIR's – since each ED has only a few cases the exclusion of one case could have a large local effect.

The GPS coordinates for cases were transformed to the Irish Grid coordinate system, overlaid onto the boundary files using R 2.15 statistical software[26] and additional packages for spatial analysis and mapping[17], [26]–[40]. The number of cases per Electoral Division was then determined. Observed cases were then divided by the expected cases based on census data to calculate crude Standardized Incidence Ratios (SIRs) per electoral division. Note that over various years of the Census, the boundaries of some Electoral Divisions have been redefined, and this necessitated the combining of some Electoral Divisions – this has been previously described by the All Ireland Cancer Atlas and we have followed the same combinations of Electoral Divisions as outlined in Appendix 2 of their report (Tables A2.1–A2.4)[16]. There were 3,355 ED's after combinations.

Next the *poly2nb()* command of R's *spdep* package[31] was used to determine neighbour relations between Electoral Divisions. Again as outlined in Appendix 2 (Table A2.5) of the All Ireland Cancer Atlas, we created artificial neighbour relations between islands, headlands and peninsulas[16]. In addition we created 2 additional artificial neighbour links (between ED33008 -> ED28030 and between ED33011 -> ED33016). This was because no data were available for Northern Island and as a result Donegal was connected to the rest of the island by only one neighbour link in two separate locations. True neighbours were given a weight of 1.0 whilst artificial neighbours were given a weight of 0.5.

### Spatial Smoothing Strategy

The data were input into OpenBUGS 3.2.2 [41] and Bayesian conditional autoregression implemented using the Besag-York-Mollié model[8], [17], [42], [43]. This model assumes a Poisson model for spatial distribution of cases, allowing for global and local random effects as follows:
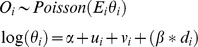
where:
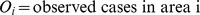


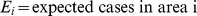


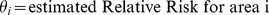


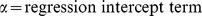


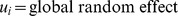


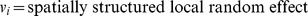


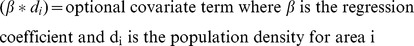



Supporting Information S1 lists the model code. The model was compiled in OpenBugs and data on observed and expected cases loaded. After initializing, OpenBugs was given a burn-in run of 1,000,000 iterations before a further production run of 1,000,000 iterations across two chains - which were thinned by 100 to yield 20,000 estimates for the Relative Risk of each ED. Convergence of the chains was assessed by graphical inspection of CODA output.

### Post Smoothing Analysis

After Bayesian smoothing, R was used to plot graphs of smoothed RR's for the entire cohort, and sub-cohorts of particular interest. Due to the skewed distribution of population density across ED's, the mean RR across ED's was 0.96. Therefore dividing by the mean was used to centre the RR's. To further examine the relationship between population density and RR, log-normalisation of the census-averaged population density per ED was attempted and uni-variate linear regression of RR's versus ln(population density) was performed for total cases and also for stratified male and female cases. The linear regression was performed separately for both smoothed RR, and smoothed RR weighted by expected cases per electoral division.

## Results

**Figure 2:**
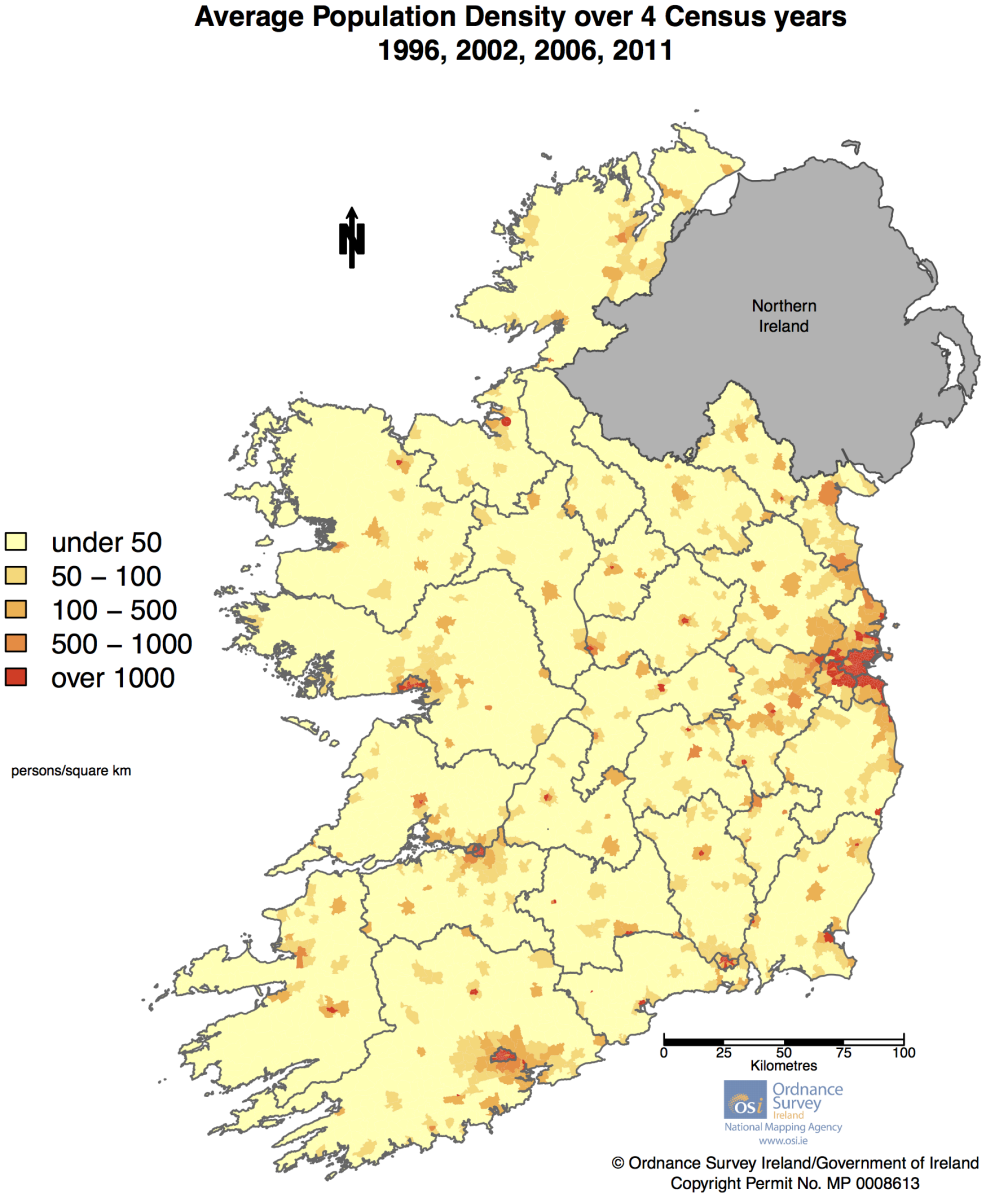
Average Population Distribution across 4 census years.

**Figure 3:**
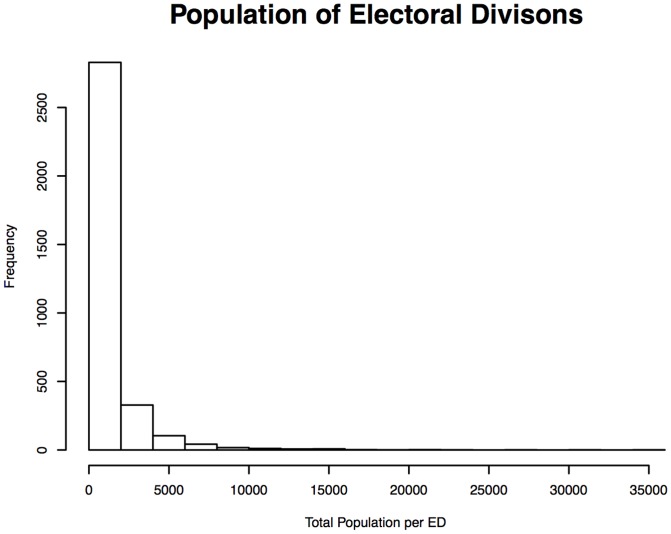
Distribution of Population per Electoral Division.

**Figure 4:**
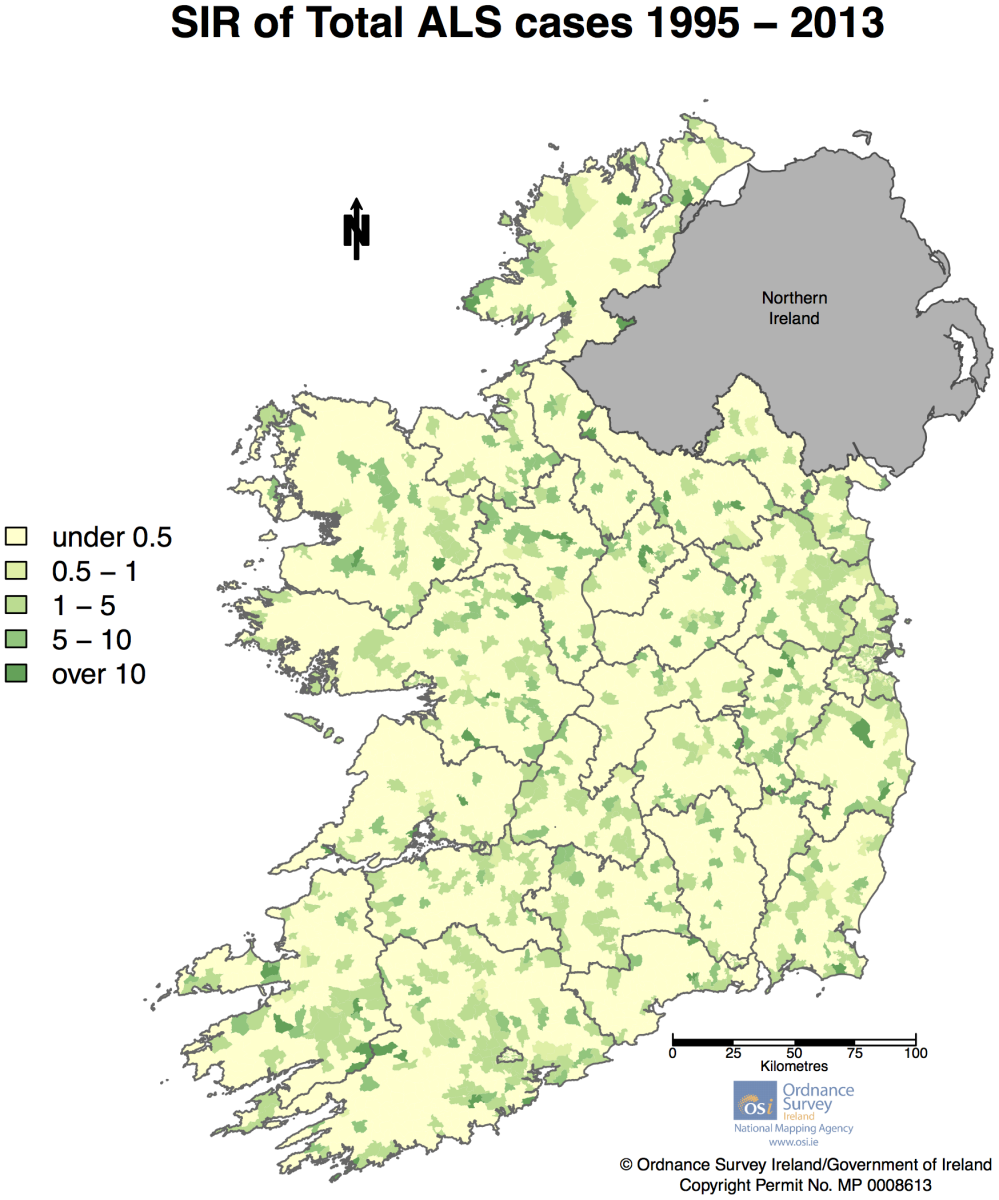
Crude (unsmoothed) standardized incidence ratio (SIR) per ED for all cases of ALS in Ireland 1995 – July 2013. Figure 4 Legend: As seen above, a large number of EDs have no cases of ALS and thus SIR  = 0. Non-zero SIR values ranged up to 24.56.

The total number of cases meeting the inclusion criteria was 1,645. Of those only 7 could not be geocoded (due to incomplete addresses) leaving 1,638 geocoded cases. Table 1 shows the clinical characteristics of the cohort. Only 61 cases had no known age at diagnosis (due either to a missing birthdate or a missing diagnosis date). The population density per ED, after averaging population across census years, is shown in figure 2. Figure 3 displays a histogram of total population per ED after census averages were calculated. As can be seen the distribution is highly asymmetric with a very large number of EDs having a population less than 2,000, and a small number of ED's with population into the tens of thousands. Figure 4 displays crude SIRs for all cases of ALS in the Republic of Ireland between January 1995 and July 2013. Due to random variation it is difficult to discern any overall geographical pattern in the map.

**Table 1:** Basic clinical characteristics of Irish ALS patients from Jan 1995 to July 2013 (N = 1,645).

Variable		Missing
Geocoded?	1638 (99.6%)	7 (0.4%)
Sex		
Male	934 (56.8%)	
Female	711 (43.2%)	0
Escorial		
Definite	876 (57.9%)	
Probable	436 (28.8%)	
Possible	183 (12.1%)	
Suspected	17 (1.1%)	133 (8%)
Site Of Onset		
Limb	923 (59.1%)	
Other	638 (40.9%)	84 (5.1%)
Age at diagnosis		
Median (range)	66.3 (21–94)	61 (3.7%)

**Figure 5:**
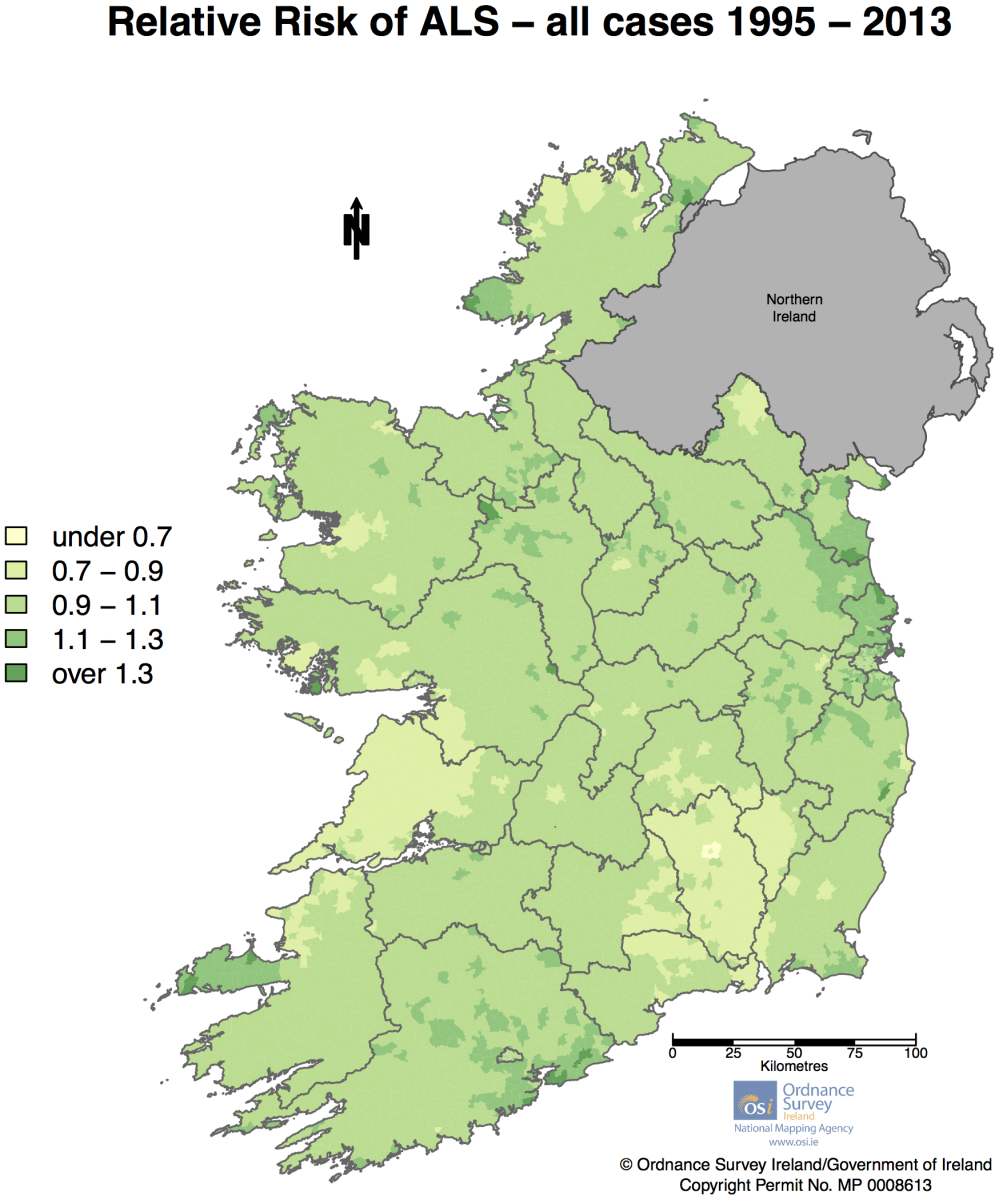
Smoothed Relative Risk for all cases of ALS in Ireland from 1995 to July 2013.

Initial Bayesian smoothing of all cases including total population density revealed a coefficient for population density that did not differ significantly from zero (mean: −1.60×10^−5^; 95% credible interval: −4.73×10^−5^ to 1.36×10^−5^). As this result indicated that population density was not associated with ALS risk, this was therefore omitted from further models. Figure 5 displays smoothed relative risks for all cases of ALS after Bayesian smoothing without including a term for population density. As can be seen there is a variation in RR across the country with RRs ranging from 0.69–1.58. Several areas appeared to have slightly higher RR – the North-East coast (Counties Louth, Meath and North County Dublin), Cork city, the Dingle peninsula in Kerry, and the Western part of Donegal. County Kilkenny appears to have lower RR, as does the Western part of County Clare.

**Figure 6:**
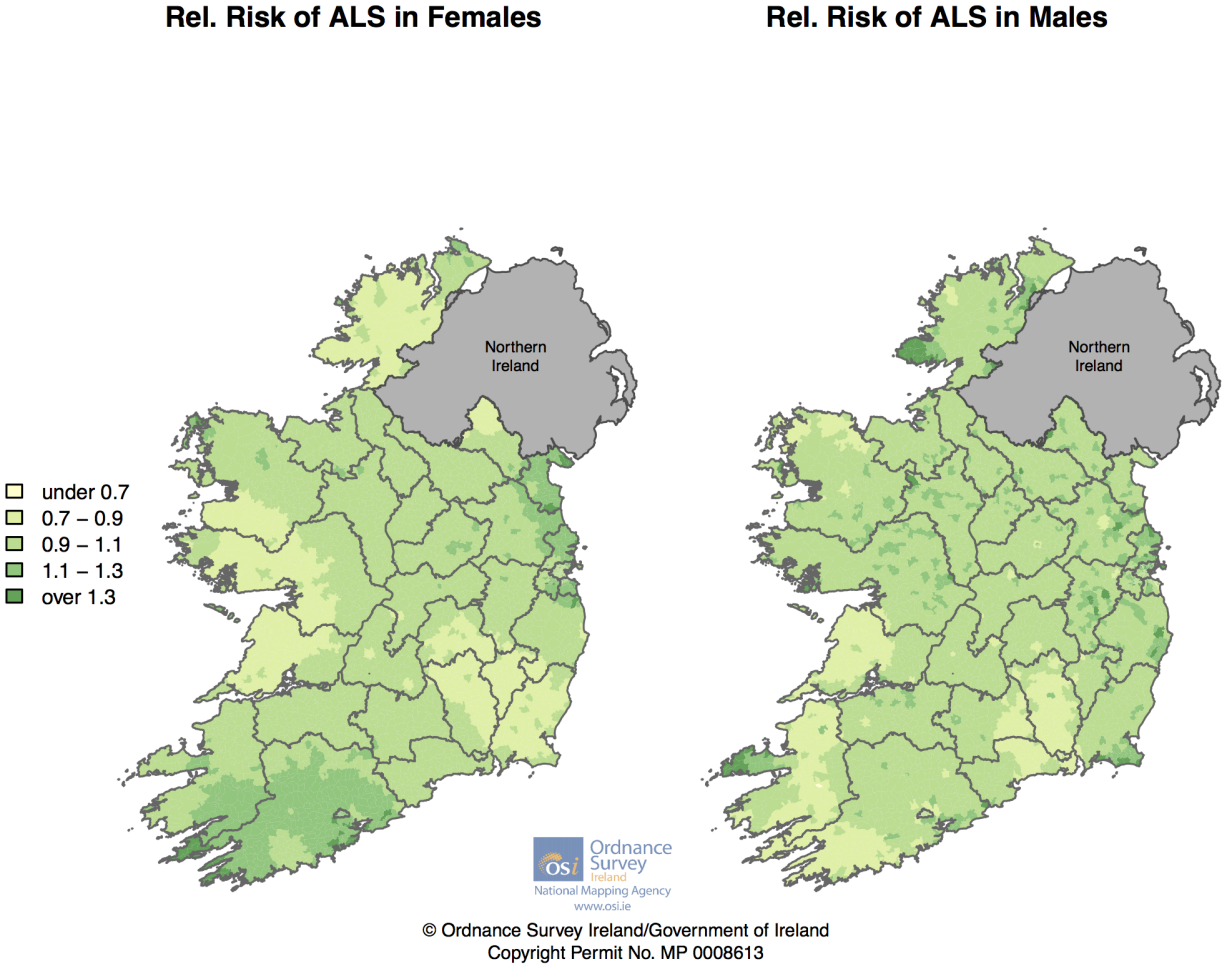
Smoothed Relative Risks for male and female Irish ALS cases 1995 – July 2013.

**Figure 7:**
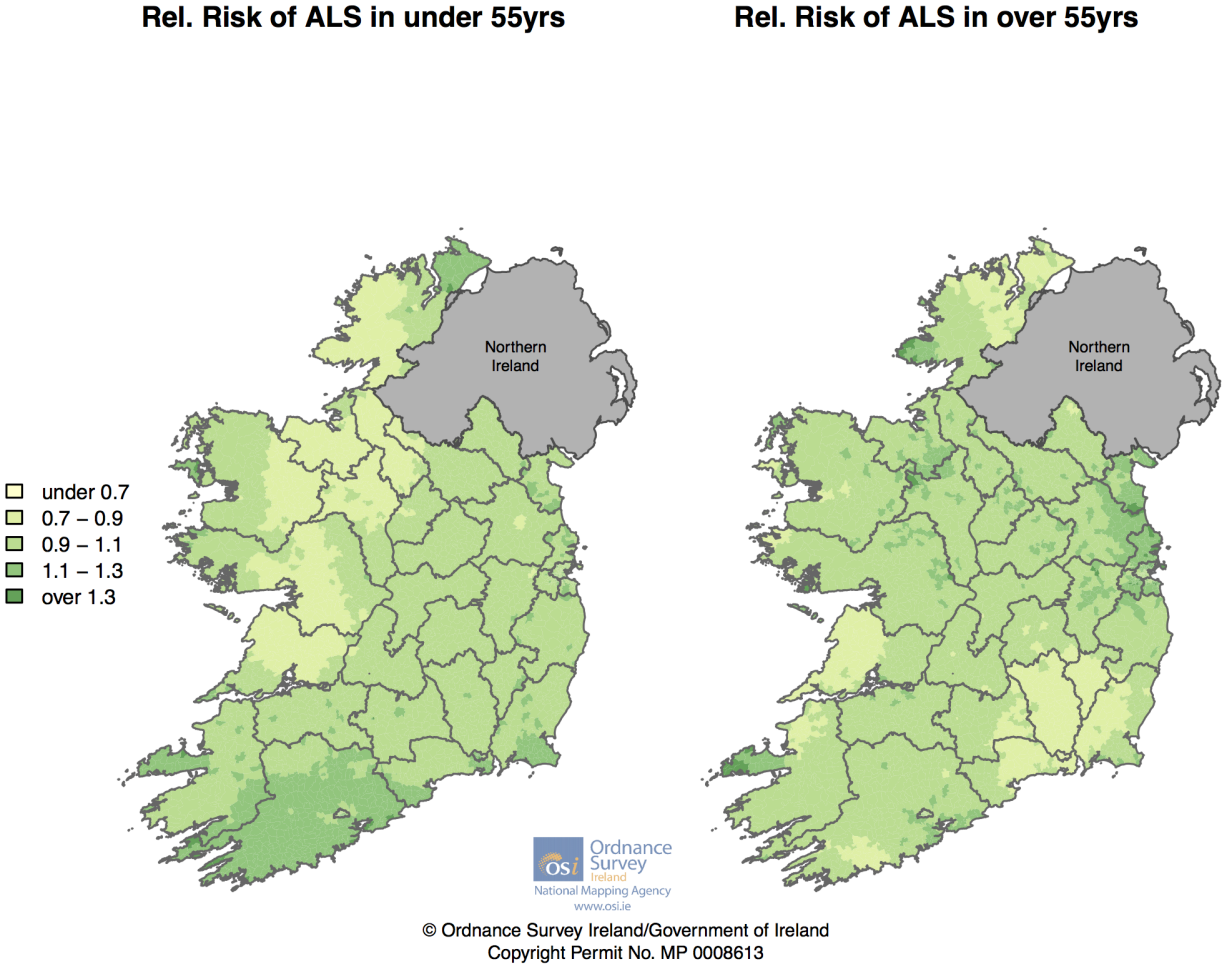
Smoothed Relative Risks for Irish ALS cases aged under 55 and over 55 at diagnosis between 1995 – July 2013.

Figure 6 displays smoothed relative risks separately for male and female cases. Both graphs show only mild spatial variation in general, however there are some localized areas of interest. For females, parts of Kerry, Cork and Louth/Meath show higher RRs, whilst for males, the Dingle peninsula and the Western-most section of Donegal show higher RR. Figure 7 displays smoothed relative risks separately for those aged under 55 and those over 55 at diagnosis (early onset ALS is more likely to be of genetic origin[2]). Those diagnosed under the age of 55 (n = 349) show a pattern of raised RR in the Cork/Kerry area, whereas those diagnosed over the age of 55 (n = 1,289) shows a raised RR in the Louth/Meath/Dublin area.

**Figure 8:**
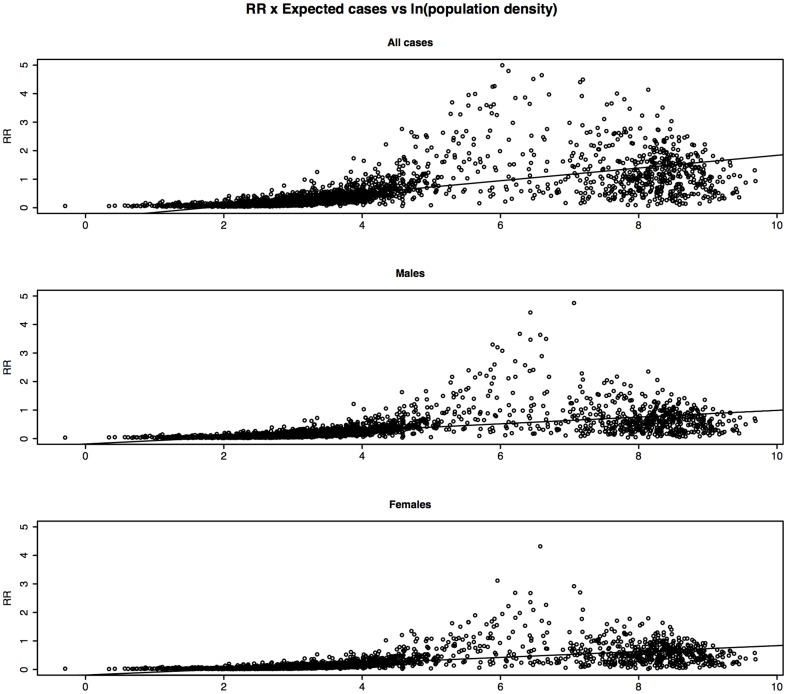
Linear Regression of post Bayesian smoothing RR x expected cases for all cases, males and females versus ln(population density). Figure 8 Legend: The regression coefficients were as follows: Weighted: All cases: Coeff.  = 0.22; 95%CI(0.211, 0.234); P<2×10^−16^; R^2^ = 0.32 Males only: Coeff.  = 0.12; 95%CI(0.112, 0.124); P<2×10^−16^; R^2^ = 0.31 Females only: Coeff.  = 0.10; 95%CI(0.098, 0.108); P<2×10^−16^; R^2^ = 0.34 Unweighted: (not shown in graph) All cases: Coeff.  = 0.016; 95%CI(0.014, 0.017); P<2×10^−16^; R^2^ = 0.10 Males only: Coeff.  = 0.011; 95%CI(0.009, 0.013); P<2×10^−16^; R^2^ = 0.04 Females only: Coeff.  = 0.018; 95%CI(0.016, 0.020); P<2×10^−16^; R^2^ = 0.11.

Graphs of RR weighted by expected cases for all cases, males and females versus natural log of population density are shown in figure 8. As can be seen there is a statistically significant relationship between the smoothed weighted RR of all cases and ln(pop. density). For the un-weighted regressions, we found a statistically significant relationship between RR and ln(pop. density) with R^2^ of 0.10, 0.11 & 0.04 for all cases, females and males respectively.

## Discussion

To the best of our knowledge, this is the first areal mapping of ALS incidence based upon a national prospective population based ALS register. The overall pattern of RRs for all cases displays mild variation throughout the country. No nationwide North-South pattern (as reported by some earlier studies[12], [14], [44]) could be discerned (figure 5). Areas of moderately higher risk appear in North Dublin, Louth & Meath, Cork, the Dingle peninsula in Kerry, and the area on the south-west coast of Donegal. Of these areas, North Dublin, Louth, Meath and Cork are relatively developed and of higher population density (figure 2), whilst the area in Kerry and Donegal includes primarily rural areas and small traditional fishing with populations that have experienced little inward immigration. These factors may suggest the possibility of local genetic founder effects in some regions – an observation supported by the increased RR for those diagnosed aged under 55 years in the Cork/Kerry area.

Our findings contrast those of other groups. A 2009 Swedish study utilizing a hospital inpatient register with partial patient histories identified 3,390 incident cases of ALS between 1991 and 2005 [44]. Subsequent analysis of areal standardized incidence rates (SIR's) suggested an increasing South to North risk gradient – although a test for linear trend was not significant[44]. Similarly, a geographical and temporal analysis of ALS mortality between 1990–2005 in Spain identified 9,475 deaths –with higher rate in the North[12]. This finding may reflect the population genetics of Spain with higher degree of admixture in the South, as a previous population based mortality study in Cuba suggests that admixed populations exhibit a lower rate of ALS[45].

A study of age adjusted ALS mortality rates from 1969–1998 that divided the US into 12 geographical areas identified 105,318 deaths due to ALS according to death certificates[14]. This study also found a broad geographic pattern with declining rates in a North-Western to South-Eastern distribution, both in the total population and also in non-Hispanic whites[14]. As the population substructure in the US differs with respect to ethnicity, this distribution may reflect subtle differences in genetic risk.

Results from Bayesian spatial auto-regression vs post-Bayesian linear regression analysis of the association of population density and RR were conflicting. This discrepancy may be routed in the asymmetrical population (Fig 3) and population density distributions across electoral divisions (Fig 2), combined with different mathematical relationships employed by the two approaches: i.e. the Bayesian approach specifies ln(RR) as a function of population density (and other variables), whereas in the linear regression we examined the relationship between RR and ln(population density) – a more symmetrical distribution. This demonstrates the mathematical problems that can be caused in areal spatial analysis by an asymmetric population distribution across areas.

Notwithstanding, our finding of a relationship between rates of ALS and population density is in keeping with 2009 findings from a register based study in the South-East of England which used Poisson spatial regression and SaTScan to find a cluster of cases in greater London[10]. The findings contrast with a 2013 Italian register based analysis performed using data from the Piedmont and Aosta Valley Register for ALS[8]. The study was methodologically similar, and identified 1,216 patients diagnosed between 1995 and 2004 before using Bayesian smoothing to create maps of RR's for subgroups of the cohort. The authors found rural areas of low population density and high ALS risk. They theorized that areas of higher incidence post smoothing might be related to the use of agricultural chemicals or clusters of familial disease in rural and mountainous areas prone to genetic isolation[8] – factors which may subtly differ between countries due to differences in geography and agricultural practices. Converse to the Italian findings, our finding of increased risk in higher population density areas points towards urban environmental effects or possibly urban socio-economic factors as risk factors.

The strengths of this study are that it uses a national prospective ALS register as the case-source. It also has a very high level of data integrity with complete data on gender, just 7 cases (0.4%) lacking geocodes and 61 (3.7%) lacking a precise age at diagnosis – therefore bias due to missing data is unlikely. We have used high quality census and spatial data obtained from the CSO and the OSI, and we have implemented a robust smoothing strategy which has previously been implemented in Irish cancer epidemiology[16].

There are however some weaknesses to the study – primarily the asymmetric distribution of population amongst Electoral Divisions. We have also performed the analysis inclusive of known familial/genetic cases of ALS that may have led to higher SIRs & RRs in certain areas. Finally we have not included a formal cluster analysis at this point – although this was not our aim in this phase of the study.

In conclusion, our spatial mapping of population based incident ALS cases from 1995 to 2013 in Ireland revealed several localized areas of higher relative risk for ALS. While we found conflicting results after using two different approaches to determine if relative risk was related to population density, an approach of linear regression of post Bayesian risk estimates found evidence of a weak relationship, suggesting the possibility of increased risk in urban rather than rural areas. Further analysis of the Irish ALS population is currently underway, including detailed cluster analysis and genetic epidemiologic mapping.

## Supporting Information

Supporting Information S1 (DOCX)
