# Supplementary material for: An Exploratory Spatial Analysis of ALS Incidence in Ireland over 17.5 Years (1995 – July 2013)
Source: PLoS One. 2014 May 27;9(5):e96556. doi: 10.1371/journal.pone.0096556 (PMC4035264; doi:10.1371/journal.pone.0096556)
Supplement: Supporting Information S1 — (DOCX) [file pone.0096556.s001.docx]

# Supporting Information S1

## Openbugs model specification (excluding population density term)

model

{

for(i in 1:N)

{

observed[i]~dpois(mu[i])

log(theta[i])<-alpha + u[i] + v[i]

mu[i]<-expected[i]*theta[i]

u[i] ~dnorm(0,tau)

}

v[1:N]~car.normal(adj[],weights[],num[],precv)

alpha ~dflat()

tau ~dgamma(0.001,0.001)

precv ~dgamma(0.1,0.1)

}
